# A Web-Based Self-Management Intervention for Veterans With Chronic Conditions and Their Caregivers: A Pilot Study

**DOI:** 10.1093/geroni/igab046.1221

**Published:** 2021-12-17

**Authors:** Ranak Trivedi, Katherine Plummer, Madhuvanthi Suresh, Rashmi Risbud, Marika Humber, Donna Zulman, Christine Timko, John Piette

**Affiliations:** 1 Stanford University, Menlo Park, California, United States; 2 Palo Alto University, Atlanta, Georgia, United States; 3 VA Palo Alto Health Care System, Palo Alto, California, United States; 4 VA Palo Alto Healthcare System, Menlo Park, California, United States; 5 VA Palo Alto Health Care System, Menlo Park, California, United States; 6 University of Michigan, Menlo Park, California, United States

## Abstract

Web-based Self-management Using Collaborative Coping Enhancement in Diseases (Web-SUCCEED) is a dyadic intervention for patients and their caregivers designed to improve self-management through improving dyadic stress coping, dyadic relationships, and positive emotions. Veterans Affairs (VA) patients with one or more chronic conditions and positive screen for self-management distress were recruited with their informal caregiver from VA Palo Alto. Of the 17 patients and 16 caregivers recruited (62.3% of eligible), 8 patients and 8 caregivers (48.5%) completed the intervention and assessments. Twelve participants withdrew mostly citing the stress of the pandemic as their reason; 5 did not respond to multiple outreach efforts. Veterans were 66□18 y and caregivers were 58□16 y. Veterans and caregivers who completed the program rated it high on usability and acceptability. Pre-post t-tests across a psychosocial battery did not reveal significant differences; results were limited by incomplete post-intervention data. Further testing with modified retention strategies is recommended.

